# Comparison of Muscle Mass Between Healthy Subjects and Patients With Malignant Tumors Undergoing Outpatient Treatment

**DOI:** 10.7759/cureus.42462

**Published:** 2023-07-25

**Authors:** Hiromitsu Takaoka, Takeo Furuya, Yasuhiro Shiga, Satoshi Maki, Kazuhide Inage, Satoshi Yamaguchi, Takeshi Yamashita, Takahisa Sasho, Hirotaka Kawano, Seiji Ohtori

**Affiliations:** 1 Department of Orthopaedic Surgery, Chiba University's Graduate School of Medicine, Chiba, JPN; 2 Department of Orthopaedic Surgery, Oyumino Central Hospital, Chiba, JPN; 3 Center for Preventive Medical Sciences, Chiba University, Chiba, JPN; 4 Department of Orthopaedic Surgery, Teikyo University School of Medicine, Tokyo, JPN

**Keywords:** malignant tumor patient, bone metastasis outpatient, trunk muscle, healthy subject, skeletal muscle mass index

## Abstract

Background

In recent years, advances in the treatment of malignant tumors have improved life expectancy and diversified treatment options. However, maintaining high activities of daily living in patients is essential for appropriately treating the primary disease, and interventions for patients with impaired motor function will lead to improved quality of life. Here, we compared the muscle mass of malignant tumor patients who are visiting bone metastasis outpatient clinics with that of healthy subjects.

Methods

We compared the muscle mass of 61 malignant tumor patients with an Eastern Cooperative Oncology Group (ECOG) performance status ≤ 2 (mean 66.3 ± 12.0 years; 30 males and 31 females) attending our bone metastasis outpatient clinic since 2018 with that of 315 healthy subjects (mean 65.0 ± 17.7 years; 110 males and 205 females). Body mass index, skeletal muscle mass, and body fat percentage were assessed by bioimpedance analysis, and the skeletal muscle mass index (SMI) was calculated.

Results

To eliminate age bias in the malignant tumor patients and healthy subjects, 1:1 propensity score matching was performed separately for males and females. There was no significant difference in right upper limb, left upper limb, right lower limb, or left lower limb mass or SMI between the two groups, whereas trunk muscle mass and muscle mass were significantly higher in the healthy females compared with malignant tumor females.

Conclusion

There was no significant difference in the SMI measured by bioimpedance analysis between the two groups in either males or females, while muscle mass and trunk muscle mass were significantly lower in female malignant tumor patients than in healthy subjects. These results suggest that even malignant tumor patients whose performance status was maintained enough to allow outpatient visits still had impaired motor function.

## Introduction

Recently, the prognosis of certain malignant tumors has been improved by multidisciplinary treatment combining surgery, chemotherapy, and radiotherapy. On the other hand, malignancy often induces metabolic changes such as anorexia, hypometabolism, or hypercatabolism, leading to changes in body composition and sometimes a decrease in skeletal muscle mass [1,2]. Skeletal muscle mass loss is often accompanied by muscle weakness and decreased physical performance, leading to sarcopenia. In addition, there are some reports that loss of skeletal muscle mass decreases the responsiveness to chemotherapy [1], and that the presence of sarcopenia is associated with survival in gastrointestinal cancer patients [3,4]. Therefore, it is important to focus on the assessment of skeletal muscle mass in patients with malignant tumors. However, most reports have been based on the measurement of trunk muscle mass area by CT [3,4]. There are few reports on the measurement of skeletal muscle mass by bioelectrical impedance analysis (BIA), and its relationship to the muscle mass of healthy subjects is not clear. In this study, we measured and compared the trunk and limb muscle mass between healthy subjects and patients with malignant tumors undergoing outpatient treatment.

## Materials and methods

In this cross-sectional study, we compared skeletal muscle mass index (SMI) between patients with visceral or hematological malignancies and healthy subjects. Chiba University's Graduate School of Medicine, Chiba, Japan approved the study (approval number: 3227). The subjects comprised 57 patients (mean age 66.3 ± 12.0 years, 28 males and 29 females) with malignant visceral or hematological malignancies who visited our outpatient clinic from 2018 to 2019. The inclusion criterion was an Eastern Cooperative Oncology Group (ECOG) performance status ≤ 2. Exclusion criteria were significant pain or paralysis, bone metastases with a risk of pathologic fracture, difficulty walking alone, and difficulty completing the questionnaire. For comparison, healthy subjects recruited at an event organized by our institute were evaluated. To eliminate age bias between the malignant tumor patients and healthy subjects, 1:1 matching of the groups by propensity score matching was performed for each sex.

Skeletal muscle mass

Muscle mass, fat proportion, and other parameters were measured by BIA using the MC-780A or MC-980A Body Composition Analyzer (Tanita Corporation, Tokyo, Japan). BIA is a method that measures the electrical properties (bioimpedance) of living tissue and determines body composition. Muscle mass was measured separately for the right and left upper limbs, right and left lower limbs, and trunk. Trunk muscle mass was calculated by subtracting the appendicular SMI from the muscle mass of the whole body. BMI was calculated as body weight divided by height squared at the time of BIA.

Statistical analysis

The cases were selected by 1:1 propensity score matching by age and BMI for malignant tumor patients and healthy subjects. Categorical variables are presented as numbers and continuous variables as means and standard deviations. The Mann-Whitney U test was used for statistical comparisons, and a p-value < 0.05 was considered significant. The statistical analyses were performed using SAS version 9.4 for Windows (Released 2013; SAS Institute Inc., Cary, North Carolina, United States).

## Results

Demographic data and propensity score matching

The total number of healthy subjects recruited was 315 (mean age 65.0 ± 17.7 years; 110 males and 205 females). As a result of matching, 54 (of 57) malignant tumor patients (Group M) and 54 (of 315) healthy subjects (Group N), with 28 males and 26 females in each group, were selected (Table 1). There was no significant difference in BMI between the two groups. A total of 94% of Group M had a performance status (PS) of 0 or 1. Surgical treatment of primary lesions was performed in 43% of male and 38% of female patients, chemotherapy in 57% and 45% of patients, and radiation therapy for localized or metastatic lesions in 21% and 18% of patients, respectively.

**Table 1:** Demographic data of each group after propensity score matching Group M: malignant tumor group; Group N: healthy group TP: total protein; Alb: albumin; PS: performance status

	Male					Female			
	Group M	Group N		p-value		Group M		Group N	p-value
Number of cases	28	28				26		26	
Age (years)	73.2±8.9	73.5±9.0				61.8±9.0		61.8±9.0	
BMI (kg/m^2^)	22.9±2.9	22.7±2.5		0.902		20.6±4.1		21.8±2.7	0.164
Past medical history of cancer treatment									
Surgical treatment of primary lesion	43%						38%		
Chemotherapy	57%						45%		
Radiation therapy for localized or metastatic lesion	21%						18%		
PS 0	12						9		
PS 1	15						15		
PS 2	1						2		
Bone modifying agent	57%						46%		
Metastasis pain	39%						23%		
TP/Alb (g/dl)	7.1±0.6/3.8±0.5						7.1±0.6/3.9±0.7		

Table 2 shows the malignancy types in malignant tumor patients. Lung cancer was the most prevalent among the males and breast cancer among the females.

**Table 2:** Types of malignant tumor in the malignant tumor patients group (Group M)

Type of malignant tumor		Male	Female
Lung cancer		11	6
Breast cancer		1	8
Uterine cancer		0	4
Malignant lymphoma		2	2
Colon cancer		3	0
Maxillary carcinoma		1	2
Pancreatic cancer		2	0
Prostate cancer		2	0
Esophageal cancer		1	1
Hepatocellular carcinoma		1	1
Gastric cancer		1	0
Submandibular adenocarcinoma		1	0
Malignant melanoma		1	0
Malignant pleural mesothelioma		1	0
Thyroid cancer		0	1
Renal cancer		0	1
		28	26

SMI

The results of each study are shown in Table 3. There was no significant difference in SMI between the two groups: 8.05 ± 1.41 kg/m^2^ for Group M and 7.75 ± 0.85 kg/m^2^ for Group N for males, and 6.16 ± 0.90 kg/m^2^ and 6.52 ± 0.72 kg/m^2^ for females, respectively. There were also no significant differences in the right upper limb, left upper limb, right lower limb, or left lower limb mass or SMI between the two groups in either males or females.

**Table 3:** Muscle mass, body fat percentage, and other parameters SMI: skeletal muscle mass index

	Male				Female		
	Group M	Group N	p-value		Group M	Group N	p-value
Right upper limb (kg)	2.51±0.45	2.39±0.33	0.188		1.62±0.31	1.73±0.21	0.065
Left upper limb (kg)	2.51±0.43	2.35±0.31	0.096		1.55±0.31	1.65±0.24	0.15
Right lower limb (kg)	8.70±1.64	8.17±1.25	0.234		5.87±0.89	6.26±0.17	0.121
Left lower limb (kg)	8.52±1.57	7.98±1.14	0.251		5.88±0.93	6.18±0.92	0.26
SMI (kg/m^2^)	8.05±1.41	7.75±0.85	0.652		6.16±0.90	6.52±0.72	0.057
Muscle mass (kg)	48.1±6.2	47.0±5.1	0.417		34.3±4.2	36.4±3.2	0.0315
Trunk muscle (kg)	25.8±4.1	26.1±2.7	0.688		19.4±2.3	20.5±1.5	0.0196
body fat (%)	19.5±7.1	18.5±5.4	0.606		25.8±9.3	26.3±6.5	0.749

Muscle mass and trunk muscle mass

Muscle mass was 48.1±6.2 kg for Group M and 47.0±5.1 kg for Group N in males, and 34.3±4.2 kg for Group M and 36.4±3.2 kg for Group N in females. In trunk muscle, Group M was 25.8±4.1 kg and Group N was 26.1±2.7 kg for males, and Group M was 19.4±2.3 kg and Group N was 20.5±1.5 kg for females. There were no significant differences in any type of muscle mass between male malignant tumor patients and male healthy subjects. On the other hand, trunk muscle mass and muscle mass were significantly higher in healthy females than in females of Group M (P < 0.05).

## Discussion

To the best of our knowledge, this is the first study to compare muscle function between outpatients with malignant tumors and healthy subjects. In this study, we showed that muscle function was decreased in female outpatients with malignant tumors compared with healthy subjects.

Demographic data

The present study had a younger average age of female malignant tumor patients compared to male patients. Peak ages of onset for breast, cervical, and uterine cancers are reported to be 55-64, 45-49, and 55-59 years, respectively. On the other hand, the incidence of lung and colon cancers, which are more common in males, is reported to increase with age, and the peak age of onset for prostate cancer is reported to be 75-79 years [5]. In other words, the younger age of incidence of malignant tumor types that predominantly affect female patients and the older age of incidence of malignant tumor types that predominantly affect males may have led to the results of this study.

SMI

There was no significant difference in the SMI measured by BIA between the two groups in either males or females. It has been reported that patients with malignant tumors and low muscle mass have a decreased quality of life, severe therapeutic toxicity, increased rates of postoperative infections and complications, increased rate of hospitalization, prolonged hospital stay, shortened survival, and poor prognosis [6,7]. Powell et al. measured preoperative muscle mass by BIA in patients with esophageal cancer undergoing surgical treatment and reported a poor prognosis in patients with low muscle mass [8]. Fukushima et al. compared muscle mass between patients with hematological tumors and healthy subjects and reported a significantly lower SMI in the patients [9]. In this study, the patient group comprised outpatients, many of whom were able to receive adequate treatment for their primary disease because of a low PS and high level of physical activity. Therefore, the results of this study were different from those of previous studies.

Muscle mass and trunk muscle mass

There were significant decreases in muscle mass and trunk muscle mass in female malignant tumor patients compared with healthy subjects. In a previous report, Fernández et al. found a significantly lower trunk muscle mass in patients with colorectal malignancies compared with healthy subjects [10]. Salhi et al. reported that trunk muscle mass was already reduced at diagnosis in patients with lung malignancies [11]. We found a similar result in females in this study, suggesting that there may be differences in muscle mass and trunk muscle mass between female healthy subjects and malignant tumor patients. When trunk muscle weakness makes it difficult to live at home, it is difficult to provide aggressive primary disease treatment. It is possible that focusing on the decline of trunk muscles may affect the duration of life expectancy. On the other hand, no significant difference was found among males. As mentioned earlier, the average age of males in this study was more than 10 years older than that of females. Therefore, it is possible that in males, trunk muscle strength declined with age even in the healthy group, and no significant difference was found. It has also been reported that aromatase inhibitors are often administered in breast cancer patients and may affect bone density loss [12]. A decrease in bone density has been reported to correlate with a decrease in muscle mass [13,14]. The use of aromatase inhibitors may also have influenced the results of this study since approximately 30% of the female patients in this study had breast cancer. Finally, some cancers are treated with platinum-based chemotherapy. This has side effects on the nervous system and may affect BIA measurements related to electrical processes in living tissue.

Limitations

There were several limitations to this study. First, it was a retrospective study. Secondly, we did not differentiate among malignant tumors, and thus muscle mass differences according to specific malignant tumors could not be investigated. Likewise, there was no differentiation in the type of treatment or the background of the subjects. Thirdly, no background information on the healthy subjects was obtained. Finally, It is possible that the number of cases was small.

## Conclusions

We compared muscle mass between outpatients with malignant tumors and healthy subjects. There was no significant difference in the SMI between the two groups in either males or females. Muscle mass and trunk muscle mass were decreased in female malignant tumor patients compared with healthy female subjects. Even in malignant tumor patients whose PS was maintained enough to allow them to visit outpatient clinics, motor function was potentially impaired. Therefore, mobility may be reduced even in patients with good PS. Interventions to maintain and improve motor function through exercise in malignant tumor patients may be necessary to treat malignant tumors and improve prognosis.
